# Common *UGT1A6* Variant Alleles Determine Acetaminophen Pharmacokinetics in Man

**DOI:** 10.3390/jpm12050720

**Published:** 2022-04-29

**Authors:** María de las Olas Cerezo-Arias, Javier Gómez-Tabales, Manuel Martí, Elena García-Martín, José A. G. Agúndez

**Affiliations:** 1 Intensive Care Medicine Department, University Hospital, Badajoz, University of Extremadura (UEX), 06006 Badajoz, Spain; maria.cerezo@salud-juntaex.es; 2 Universidad de Extremadura, University Institute of Molecular Pathology Biomarkers, ARADyAL Instituto de Salud Carlos III, 10071 Caceres, Spain; jagomezta@unex.es (J.G.-T.); mmartia@unex.es (M.M.)

**Keywords:** acetaminophen, pharmacokinetics, UGT1A6, UGT1A9

## Abstract

Acetaminophen (paracetamol) is a widely used drug that causes adverse drug events that are often dose-dependent and related to plasma drug concentrations. Acetaminophen metabolism strongly depends on UGT1A enzymes. We aimed to investigate putative factors influencing acetaminophen pharmacokinetics. We analyzed acetaminophen pharmacokinetics after intravenous administration in 186 individuals, and we determined the effect of sex; body mass index (BMI); previous and concomitant therapy with UGT1A substrates, inhibitors, and inducers; as well as common variations in the genes coding for UGT1A1, UGT1A6, and UGT1A9. We identified sex and *UGT1A6* genetic variants as major factors influencing acetaminophen pharmacokinetics, with women showing lower clearance (*p* < 0.001) and higher area under the plasma drug concentration-time curve (AUC) values than men (*p* < 0.001). *UGT1A6* genetic variants were related to decreased acetaminophen biodisposition. Individuals who were homozygous or double-heterozygous for variant *UGT1A6* alleles showed a 22.5% increase in t_1/2_ values and a 22.8 increase in drug exposure (*p* < 0.001, and 0.006, respectively) after correction by sex. The effect is related to the UGT1A6*2 and UGT1A6*4 variant alleles, whereas no effect of UGT1A6*3 and UGT1A9*3 alleles, BMI, or drug–drug interaction was identified in this study. We conclude that sex and *UGT1A6* variants determine acetaminophen pharmacokinetics, thus providing evidence to eventually developing pharmacogenomics procedures and recommendations for acetaminophen use.

## 1. Introduction

Acetaminophen (paracetamol) is a non-competitive reversible inhibitor of cyclooxygenase (COX) enzymes [1], which does not belong to the non-steroidal anti-inflammatory drugs (NSAID) group. Acetaminophen is widely used for the treatment of fever and pain [2], and it causes fewer cardiovascular, gastric, and renal adverse events than NSAIDs [3]. However, it shares with NSAIDs their antipyretic and analgesic properties as well as adverse drug events, such as hypersensitivity events in their two main clinical forms (selective hypersensitivity and cross-hypersensitivity) [4,5,6,7]. In addition, acetaminophen causes idiosyncratic drug-induced liver injury (iDILI) [8,9,10], as NSAIDs do, and it has been hypothesized that functional genetic variations in the COX-enzymes are related to iDILI risk and cross-hypersensitivity to COX-inhibitors [11,12,13,14,15], thus suggesting that patients with impaired COX activity are more prone to developing adverse drug events when using COX inhibitors [14,15]. Acetaminophen is also related to dose-dependent acute liver failure. The pathogenesis of high-dose-related acetaminophen liver failure involves the formation of the highly reactive N-acetyl-p-benzoquinone imine (NAPQI) metabolite, a product of cytochrome P450 enzymes that causes glutathione depletion and that binds covalently to proteins [16]. It has been postulated also that NAPQI could be related to selective hypersensitivity to acetaminophen [4]. Therefore, the relevance of acetaminophen metabolism is linked both to the generation of reactive metabolites and interindividual variability in acetaminophen biodisposition. The fact that about 90% of acetaminophen is metabolized in vivo supports the hypothesis that altered metabolism might lead to altered pharmacokinetics and drug response [17].

The role of UDP Glucuronosyltransferase Family 1 Member A (UGT1A) enzymes in the initial steps of acetaminophen metabolism is crucial. Acetaminophen glucuronides represent about half of the metabolites recovered in urine. The most relevant among these UGTA1 enzymes at therapeutic concentrations is UGT1A6, followed by UGT1A9 [18]. In vitro evidence suggests that acetaminophen glucuronidation is altered by *UGT1A* gene variants (that is, *UGT1A* genes with sequences different from the wild-type sequence) [18]. Therefore, it could be hypothesized that *UGT1A* gene variants might have an in vivo effect, modifying acetaminophen pharmacokinetics and hence effects. However, studies conducted so far failed to identify *UGT1A* gene variants statistically related to acetaminophen pharmacokinetics [17,18,19,20,21]. This hampers the use of pharmacogenomics information for acetaminophen. Interestingly, one of these previous studies shows a non-significant trend to lower acetaminophen biodisposition in carriers of *UGT1A* gene variants, which suggests that more powered studies might identify significant associations. For instance, a lower acetaminophen clearance in carriers of the genotype UGT1A6*2/*2 as compared with individuals with the UGT1A6*1/*1 genotype has been reported although the cohort was small (*n* = 69), and the findings did not reach statistical significance [17].

A Clinical Pharmacogenomics Implementation Consortium (CPIC) guideline for NSAID use has been already published [22]. Although acetaminophen is not considered an NSAID, it is frequently used instead of NSAIDs because of its antipyretic and painkiller properties but, due to the lack of information on significant associations, the CPIC genes-drugs website lack information on genes related to acetaminophen (see https://cpicpgx.org/genes-drugs/ (accessed on 21 March 2022)). Such information is crucial to evaluate the need for the development of clinical practice guidelines for the use of preemptive genotyping before drug use [23].

This study aims to gain ground on the putative role of common functional *UGT1A1*, *UGT1A6*, and *UGT1A9* gene variation in acetaminophen pharmacokinetics. To this end, we analyzed acetaminophen pharmacokinetics and common functional *UGT1A* gene variations in a large cohort of well-characterized participants after administration of 1 g acetaminophen. To avoid confounders related to drug absorption or first-pass biodisposition, the study group only included patients who received acetaminophen by intravenous infusion.

## 2. Patients and Methods

One hundred and ninety-three patients (125 women and 68 men) who were admitted to the Intensive Care Unit (ICU), University Hospital, Badajoz, Spain, participated in the study. Characteristics of the participants are shown in Table 1.

All participants were Spanish individuals who self-reported as Caucasians. All patients were admitted after traumatology surgery. All participants provided signed informed consent and the study was conducted according to the guidelines of the Declaration of Helsinki and approved by the Institutional Ethics and Biosecurity Committee of the University of Extremadura, Badajoz, Spain. To account for potential confounders, information on drug therapy fourteen days before study initiation was recorded to identify patients who received long-term or intermittent administration of substances known to be substrates, inhibitors, or inducers of UGT1A1, UGT1A6, or UGT1A9 enzymes according to the DrugBank online database (https://go.drugbank.com (accessed on 15 March 2022). Such information is shown in Supplementary Table S1. Besides therapy before admission to the ICU, data regarding diagnosis, sex, age, weight, body mass index, and antecedents of liver or renal disorders were recorded. In addition, concomitant drug therapy received in the ICU was accounted for in order to identify putative confounders for acetaminophen pharmacokinetics (Supplementary Table S2). Seven participants had antecedents of nephropathy or hepatopathy, and since these pathologies could modify the pharmacokinetics of acetaminophen, these patients were removed from the study, leaving 186 valid participants.

All participants received 1 g acetaminophen diluted in 100 mL saline serum (0.9% sodium chloride), with an infusion time of 15 min. Serum samples were collected every 30 min starting from the administration until 6 h after the treatment was initiated and immediately frozen at −80 °C until analysis. Sample collection was finished after 6 h because most patients received another dose of acetaminophen at that time. The protocol also included the collection of whole blood samples for genetic analyses.

Determination of acetaminophen concentration and pharmacokinetic analysis: For each sample, 10 μL serum was gently mixed with 800 μL methanol and 0.2% formic acid containing 5 μM acetaminophen-d4 (Sigma-Aldrich, Madrid, Spain), which was used as internal standard. The samples were mixed for 15 s and kept on ice for 20 min. Then, the samples were centrifuged for 10 min at 3000× *g*, at 4 °C. The supernatant (750 μL) was evaporated under a nitrogen stream. Then, the samples were resuspended in 40 μL of a mixture containing water (97.49%), methanol (2.5%), and formic acid (0.1%). The same process was used for the calibration curves using blank serum spiked with acetaminophen at concentrations ranging from 0.1 to 35 mg/L. The supernatant (10 μL) was injected onto a Synergi™ 4 µm Fusion-RP 80 Å, LC Column 50 × 2 mm (Phenomenex, Alcobendas, Spain) using a Hitachi LaChrom Elite^®^ HPLC system (Hitachi High—Technologies Corporation, Tokyo, Japan), composed by HTA L-2130 LaChrom Elite quaternary pumps, L-2200 LaChrom Elite autosampler, 5310 Chromaster column heater, and a triple quadrupole MS/MS detector API400™ (SCIEX, Alcobendas, Spain). The column temperature was set at 20 °C. The flow rate was 0.2 mL/min, and the mobile phase consisted of the following gradient: Mobile phase A: 0.1% formic acid in water; Mobile phase B: 0.1 formic acid in methanol. The elution was started with a proportion of 97.5% A:2.5% B. A linear gradient increase from 2.5% to 65%B over 0.6 min was used, and then, the mobile phase reversed to initial conditions. The mass spectrometer was operated in positive electrospray ionization using multiple reaction monitoring (MRM). The source was set at 500 °C and 5.5 kV. The target mass-to-charge ratios (*m*/*z*) for acetaminophen and deuterated acetaminophen were 152→110.2, 93.2 and 156→114.2, 97. The interday assay coefficient of variation was lower than 5%, and the intraday coefficient of variation was lower than 4%. Pharmacokinetic analyses were carried out by use of WinNonlin^®^ V5.3 (Pharsight Corporation, St. Louis, MO, USA). The pharmacokinetic model chosen to calculate all parameters was noncompartmental with intravenous infusion. Untransformed data were used for all analyses.

Genetic analyses were carried out in genomic DNA obtained from blood samples. We analyzed *UGT1A1*, *UGT1A6*, and *UGT1A9* functional variants that occur in South European populations according to the public database gnomAD (https://gnomad.broadinstitute.org/ (last accessed on 18 March 2022). For *UGT1A1,* we included in the analyses the signature single-nucleotide variants (SNVs) for UGT1A1*6 (rs4148323 G > A; Gly71Arg) and UGT1A1*27 (rs35350960 C > A; Pro229Gln). For *UGT1A6,* we analyzed four SNVs that, when combined, allow us to discriminate the variant alleles UGT1A6*2, *3, *4, *5, *8, and *9. These are rs6759892 (T > G; Ser7Ala), rs1105880 (A > G; Leu105Leu), rs2070959 A > G (Thr181Ala), and rs1105879 A > C (Arg184Ser). The synonymous SNV rs1105880 was included to facilitate *UGT1A6* allele disambiguation, which was carried out according to the *UGT1A* haplotypes and SNPs tables (https://www.pharmacogenomics.pha.ulaval.ca/ugt-alleles-nomenclature/ (last accessed on 21 March 2022). For *UGT1A9*, we analyzed the SNV rs72551330 T > C (Met33Thr), which is the signature SNV for the UGT1A9*3 allele. Other missense SNVs that have minor allele frequencies in Southern European populations equal to or lower than 0.001 according to the gnomAD database (https://gnomad.broadinstitute.org/ (last accessed on 21 March 2022) were not included in the analyses. These were: rs200903749 (UGT1A1 Ile322Val, UGT1A6 Ile321Val, and UGT1A9 Ile319 Val); rs34946978 (UGT1A1 Pro364Leu, UGT1A6 Pro363Leu, and UGT1A9 Pro361Leu); rs151238339 (UGT1A9 Val167Ala); rs35003977 (UGT1A1 Val225Gly); and rs114982090 (UGT1A6 Pro450leu). The *UGT1A1* promoter genotype was not included in the analyses because of DNA shortage. Additionally, it has been shown that the *UGT1A1* promoter genotype has no effect on acetaminophen metabolism [19]. All genotyping analyses were carried out by using TaqMan probes in triplicate in a 7500 Real-Time PCR System (Thermo Scientific, Alcobendas, Spain). Details of the TaqMan probes are shown in Supplementary Table S3. Alleles lacking the variants mentioned above were classified as *1 (non-mutated alleles) according to standard procedures.

Statistical analysis: All statistical analyses were done by using the statistical software IBM® SPSS® Statistics 19 (IBM, Armonk, NY, USA). The Mann–Whitney U and the Fisher’s test, as adequate, were used to compare age, weight, body mass index (BMI), and antecedents of nephropathy and hepatopathy. The statistical significance of the differences in pharmacokinetic parameters was calculated by use of the likelihood ratio test (LRT), including crude, and adjusted by sex values. Because pharmacokinetic parameters are strongly dependent on themselves, corrections for multiple comparisons were not adequate. We included correction by sex since our findings demonstrate that sex is a relevant factor for acetaminophen pharmacokinetics Differences were significant when *p*-values were less than 0.050.

## 3. Results

### 3.1. Interindividual Variability in Acetaminophen Pharmacokinetics

Acetaminophen pharmacokinetics displayed a marked interindividual variability, as shown in Table 2. It is to be noted that half-life (t_1/2_) values displayed a range from less than 0.5 h up to more than 3.5 h (7.5-fold), thus suggesting that large variability in the ability to eliminate acetaminophen exists. Interindividual variability in AUC values is even higher. We analyzed the influence of sex and body mass index on acetaminophen pharmacokinetics, and the results are summarized in Table 3 and Supplementary Table S4, respectively. Regarding sex, Table 3 shows statistically significant differences in C0 values (higher in women), clearance (higher in men), and AUC (higher in women). However, there are no significant differences in the t_1/2_ values. Therefore, sex-related differences seem to be related to a higher acetaminophen concentration and hence drug exposure in women. These are probably due to sex-related differences in the distribution volume, which is much lower in women than in men, as shown in Table 3. Supplementary Table S4 shows the absence of statistically significant differences when participants are classified according to their body mass indexes, which is expected due to the low liposolubility of acetaminophen [24].

To account for the putative effect of drug–drug interactions in acetaminophen pharmacokinetics, the 186 valid participants were subdivided into three groups concerning the drug therapy received within two weeks before the study initiation. The reference group was composed of patients who did not receive any substrates, inhibitors, or inducers of UGT1A1, UGT1A6, or UGT1A9 enzymes according to the DrugBank online database (https://go.drugbank.com (last accessed on 1 March 2022)).

Another group was composed of participants who received substrates of any of the mentioned enzymes, including acetylsalicylic acid (AAS), atorvastatin, buprenorphine, carvedilol, diclofenac, estradiol, furosemide, gemfibrozil, ibuprofen, indomethacin, levothyroxine, losartan, lovastatin, naproxen, acetaminophen, raloxifene, simvastatin, or tramadol. The third group was composed of participants who received inhibitors of UGT1A1, UGT1A6, or UGT1A9 enzymes, including amitriptyline, diflunisal, gemfibrozil, or indomethacin. It is to be noted that some drugs are both substrates and inhibitors for UGT1A1, UGT1A6, or UGT1A9 enzymes, and therefore, some patients are present both in the group of those who received substrates and those who received inhibitors. There were no participants receiving UGT1A1, UGT1A6, or UGT1A9 inducers according to Supplementary Table S1. The pharmacokinetic parameters for these three groups are summarized in Table 4.

Since sex-related differences for some pharmacokinetic parameters were observed (see above), we included adjustment for sex in the comparison values. No statistically significant differences in the pharmacokinetic parameters were observed when comparing the three groups of patients regarding drug therapy before the study initiation. In fact, t_1/2_ and AUC values were slightly lower in patients receiving substrates or inhibitors than in those who did not. This suggests that drug therapy before the study initiation (limited to the drugs and the doses shown in Supplementary Table S1) did not significantly affect acetaminophen pharmacokinetics. We also analyzed the putative effect of drug therapy administered concomitantly to acetaminophen (Supplementary Table S2). To that end, we classified the 186 valid participants into three categories: The first subgroup was composed of participants who did not receive any substrates, inhibitors, or inducers of UGT1A1, UGT1A6, or UGT1A9 enzymes (reference group). The second subgroup was composed of patients who received substrates for any of these enzymes, including AAS, atorvastatin, carvedilol, furosemide, morphine, propofol, tramadol, or valproic acid. Finally, the third category was represented by a single patient who received inhibitors (valproic acid and propofol), and this patient was also the only one who received inducers (carbamazepine and rifampicin). The pharmacokinetic results for these two subgroups and this single patient (under the category “other”) are shown in Table 5. The t_1/2_ values were slightly higher in patients who received UGT1A substrates, but the differences were not statistically significant. Moreover, AUC values were lower in patients who received UGT1A substrates, thus suggesting that acetaminophen pharmacokinetics is not significantly affected in patients receiving the UGT1A1, UGT1A6, or UGT1A9 substrates, listed in the Supplementary Table S2, at the doses used in this study.

### 3.2. Genetic Analyses

We did not identify any carriers of the *UGT1A1* variants rs4148323 G/A or rs35350960 C/A. This is consistent with the low minor allele frequencies described for these SNVs in Southern Europeans (see Supplementary Table S3). Therefore, as expected, *UGT1A1* variants were not informative in this study. Regardless of the low allele frequency in the South European population, we decided to test for these variants aiming to identify individuals with rare genotypes who could alter the results for the putative association of other SNVs and acetaminophen pharmacokinetics. We identified carriers of the rest of the SNVs tested for *UGT1A6* and *UGT1A9,* and in all cases, these SNVs were in Hardy–Weinberg’s equilibrium, and their allele frequencies corresponded to those described in gnomAD for Southern Europeans. Genotyping results are summarized in Supplementary Table S3.

To analyze the effect of *UGT1A6* and *UGT1A9* genotypes in acetaminophen pharmacokinetics and to avoid that the pharmacokinetics findings for a genotype might be influenced by the other one, in the study of the effect of *UGT1A6,* we excluded from the 186 valid participants all who carried *UGT1A9* variants (*n* = 11). We stratified the remaining 175 participants according to their *UGT1A6* genotypes, that is, according to the number of mutated *UGT1A6* alleles. The findings are shown in Table 6.

Individuals with one *UGT1A6* mutated gene in combination with a UGT1A6*1 gene displayed similar pharmacokinetic parameters as individuals homozygous for UGT1A6*1/*1. In contrast, individuals with two *UGT1A6* mutated genes displayed significant differences related to the elimination constant, t_1/2_, AUC, and MRT values. The differences were greater when the comparisons were corrected by sex. Our findings indicate that individuals homozygous or double heterozygous for variant *UGT1A6* alleles show increased t_1/2_ values (22.5% increase) and increased drug exposure (22.8% increase in AUMC) as compared with individuals with the UGT1A6*1/*1 genotype.

To further elucidate the putative effect of specific *UGT1A6* variant alleles, we analyzed acetaminophen pharmacokinetics stratifying participants according to their *UGT1A6* diplotypes. Results are shown in Table 7. Rare diplotypes were grouped in the category UGT1A6*1/*other and included UGT1A6*1/*4 and *1/*9. Significant differences were observed when comparing the elimination constant, t_1/2_, AUC, and MRT values for carriers of UGT1A6*2/*2 or *2/*4 diplotypes versus individuals with the UGT1A6*1/*1 diplotype. Our findings indicate that subjects carrying UGT1A6*2/*2 or *2/*4 diplotypes show increased t_1/2_ values (38% and 31%, respectively) and increased drug exposure (66% and 76% increase in AUMC, respectively). Again, the differences were greater when the comparisons were corrected by sex. Differences were observed in heterozygous carriers of these allelic variants, too, although the effect size was lower, and it was insufficient to reach statistical significance. The findings are shown in Table 7 and point to UGT1A6*2 and UGT1A6*4 as the alleles with a greater effect, whereas UGT1A6*3 seems to have a lesser effect if any.

To analyze the putative effect of *UGT1A9* genotypes on acetaminophen pharmacokinetics, we excluded all patients who carried *UGT1A6* gene variants, and hence, the comparison was limited to 65 individuals. Only four individuals with heterozygous UGT1A9*1/*3 and one with homozygous UGT1A9*3/*3 were identified in this subgroup of participants. No major differences in the pharmacokinetics of acetaminophen were observed (see Supplementary Table S5) although the number of carriers of UGT1A9*3 is too small to obtain conclusive evidence. To analyze the putative effect of *UGT1A6* and *UGT1A9* genotypes combined, we stratified participants into three subgroups: individuals with no *UGT1A6* or *UGT1A9* variant alleles, individuals with a single variant allele in either gene, and individuals with two or more variant alleles combining both genes. Statistically significant differences were observed in individuals with two or more variant alleles when compared to those with no variant alleles. However, the differences were similar to those shown for *UGT1A6* genotypes only (Table 6), thus suggesting that the effect is due to *UGT1A6* gene variations rather than to the combination of *UGT1A6* and *UGT1A9*. This is consistent with the fact that at therapeutic concentrations, UGT1A6 is more relevant in acetaminophen metabolism than UGT1A9 [18].

Since the findings obtained in this study clearly show an effect of *UGT1A6* genotypes in acetaminophen pharmacokinetics, we analyzed whether the need for on-demand opiate painkiller therapy was related to acetaminophen pharmacokinetics, that is, whether individuals who required opiates (morphine, tramadol, or fentanyl) displayed different pharmacokinetics profiles than those who did not. A single participant did not require additional painkiller therapy, 38 subjects were treated with NSAIDs only, and 147 required opiates. No significant differences were identified, as shown in Supplementary Table S6. We also checked whether *UGT1A6* and *UGT1A9* genotypes were related to the need for on-demand opiate painkiller therapy. Results are shown in Supplementary Table S7 and do not support any association. None of the participants developed adverse drug events attributable to acetaminophen, and therefore, the putative association of acetaminophen pharmacokinetics with adverse drug events could not be assessed in this study.

## 4. Discussion

Acetaminophen is extensively metabolized in humans, and large inter-individual variability in acetaminophen metabolism exists [17]. The first metabolic pathways include glucuronidation by several UGT enzymes, and among these, the most relevant is UGT1A6 [18]. Acetaminophen glucuronides account for more than 50% of the urine metabolites [4]. Unlike NSAIDs, acetaminophen causes frequent dose-dependent toxicity [25], and it is plausible that, if genetic variations related to impaired acetaminophen biodisposition could be identified, impaired acetaminophen metabolism could be a relevant pharmacogenomics target. However, evidence supporting an unambiguous relationship between acetaminophen pharmacokinetics and polymorphisms in genes coding for acetaminophen-metabolizing enzymes has not been provided so far.

The main UGT1A enzyme in acetaminophen metabolism at therapeutic doses seems to be UGT1A6, followed by UGT1A9 and UGT1A1 [26]. For that reason, several studies have been carried out attempting to investigate the putative role of common functional polymorphisms in the genes coding for these enzymes in acetaminophen pharmacokinetics and/or adverse drug events. Unfortunately, no strong associations have been identified so far. Regarding *UGT1A1*, a recent study carried out in Japanese individuals analyzed two allelic variants, UGT1A1*6 (rs4148323, a missense variant) and UGT1A1*28 (a VTR in the promoter region), in individuals receiving 3 g acetaminophen per day for 28 days and revealed that carriers of variant alleles had increased susceptibility to presenting increased alanine aminotransferase levels [27]. Although UGT1A1*6 has been related to decreased enzyme activity [28], this variant is extremely rare in individuals of European descent. We included this variant in our study (Supplementary Table S3), but we could not identify any carrier of this variant. As for the UGT1A1*28 allelic variant, we did not include this variant in the study because it has been shown that it has no effect on acetaminophen biodisposition [19]. By turn, we included the signature SNV for the UGT1A1*27 allele (rs35350960) because it causes an amino acid substitution (Pro229Gln), which is predicted as possibly damaging by Polyphen (http://genetics.bwh.harvard.edu/pph2/ (accessed on 16 March 2022)). However, such variant allele is extremely rare in individuals of European descent as shown in Supplementary Table S3, and no carriers of such variant were identified in the study group. Concerning *UGT1A9*, we tested the common variant allele UGT1A9*3, which has a functional effect, and it is related to exposure to other drugs, such as raltegravir [29]. As for *UGT1A6*, several variant alleles have been described, but information on their effect on acetaminophen metabolism is scarce. In vitro studies indicate that the variant allele UGT1A6*2 causes increased enzyme activity towards acetaminophen and other substrates [30]. These findings were partially supported by another study that reported a lower concentration of acetaminophen in urine for carriers of the UGT1A6*2 alleles as compared to carriers of non-mutated *UGT1A6* alleles although the statistical significance of such finding was marginal [21]. Regarding pharmacokinetic studies, decreased acetaminophen glucuronidation clearance has been described in carriers of UGT1A6*2 alleles, with a 15% and a 22% reduction in the clearance for heterozygous and homozygous individuals, respectively, although the differences were not statistically significant [17]. These results are quite interesting since they point to the opposite effect as that observed in vitro; that is, UGT1A6*2 is related to decreased activity in vivo. In such a study, carriers of the UGT1A6*2 allele have also a lower ratio of glucuronide/acetaminophen as non-carriers (roughly 17% in heterozygous and 27% in homozygous individuals). Although the effect of UGT1A6*2 in such a study [17] points to the same effect as that observed in our study, the effect size in our study is higher (Table 7). Putative factors influencing differences between the effect size observed in the present and the previous study are that the previous study included only 94 individuals (about half than the present study) and that acetaminophen was administered *per os*. Oral administration makes it difficult to ascertain acetaminophen pharmacokinetic parameters because of the plethora of factors influencing drug absorption [31]. In addition, the previous study did not include *UGT1A6* variant alleles other than UGT1A6*2. Besides the use of intravenous administration and the sample size, strong points in the present study include the assessment of previous and concomitant therapy and the separate analyses of the effect of variants in one gene (*UGT1A6* or *UGT1A9*) in individuals lacking common variants in the other gene or the *UGT1A1* gene. This design avoids confounders and makes the findings more reliable than in previous studies. The most relevant finding in this study is that some *UGT1A6* variant alleles are strongly related to acetaminophen pharmacokinetics. These findings point to a reduced metabolic capacity of the variant UGT1A6*2 and *4 gene products in vivo. This raises the question of whether preemptive UGT1A6 genotyping might be of use to predict acetaminophen pharmacokinetics and lead us to speculate whether it could be useful to predict drug accumulation in multiple-dosing administration and/or the occurrence of adverse drug events with acetaminophen. Much additional work is needed to answer these questions, but this study provides a functional basis for such additional research.

Besides *UGT1A6* polymorphisms, other factors influencing acetaminophen glucuronidation are sex ([17] and this study), concomitant therapy [32], and the influence of miRNAs [33]. Regarding sex, a shorter although not statistically significant acetaminophen t_1/2_ for women as compared to men has been reported [17]. In this study, we observed the same effect (Table 3), and the differences were non-significant also. The rest of the sex-related pharmacokinetic differences displayed in Table 3 were not analyzed in any previous study. Concerning concomitant therapy, our findings (Table 4 and Table 5) show the absence of relevant pharmacokinetic effects when patients were treated with UGT1A substrates, inducers, or inhibitors at the doses shown in Supplementary Tables S1 and S2. Therefore, we can conclude that, in this study, *UGT1A6* genotyping is far more predictive of acetaminophen pharmacokinetics than drug–drug interaction. Regarding acetaminophen effects, we assessed the need for painkiller therapy, and we did not identify any association either with pharmacokinetics or with *UGT1A6* genotypes. It should be kept in mind, however, that the need for painkiller therapy was greatly influenced by the cause for admission to the ICU and by additional factors, including pain sensitivity; therefore, the lack of a significant relationship does not rule out that such a relationship might actually exist.

Limitations in this study include that acetaminophen pharmacokinetics was determined after a single dose, and therefore, we could not assess the effect size in multiple administration therapy. Furthermore, we could not identify carriers of *UGT1A1* gene variants, and the number of carriers of UGT1A9*3 was limited after removing carriers of *UGT1A6* variants, and therefore, we could only ascertain the effect of *UGT1A6* variants. We could not assess any putative relationship of acetaminophen pharmacokinetics or *UGT1A6* genotypes with adverse drug events because no adverse drug events attributable to acetaminophen were experienced by any of the participants. However, previous genetic analyses of *UGT1A1*, *UGT1A6*, and *UGT1A9* among other genes involved in acetaminophen metabolism failed to identify any association with the risk of developing acetaminophen-induced acute liver failure [34].

## 5. Conclusions

In summary, our findings support a statistically significant influence of *UGT1A6* variant alleles, particularly UGT1A6*2 and UGT1A6*4, on acetaminophen pharmacokinetics, with carriers of variant alleles having a higher drug exposure and with a gene–dose effect. The effect size for pharmacokinetic changes could be considered moderate, but it should be taken into consideration that this study was carried out with a single dose of acetaminophen and that the pharmacokinetic effects are likely to be higher in multiple-dose administration. Another conclusion in this study is that the *UGT1A6* genotype is far more informative for predicting acetaminophen pharmacokinetics than UGT1A9*3 genotyping. This is obviously influenced by the allele frequency, which in the population studied here is relatively low. The frequency for UGT1A9*3 in Europeans is, however, the highest across all major human populations, and therefore, clarification of the effect of UGT1A9*3 on acetaminophen metabolism in other populations would need very large sample sizes, especially if individuals with *UGT1A6* and *UGT1A1* gene variants were discarded to avoid a confounding effect.

## Figures and Tables

**Table 1:** Characteristics of the participants.

Characteristics	Men	Women	Total	Intergroup Comparison Values
No. (%)	68 (32.6%)	125 (64.8%)	193 (100%)	-
Age (years): Mean; median, range	63.6; 65, 25–83	68.2; 71, 17–85	66.6; 70, 17–85	0.004
Weight (kg): Mean; median, range	84.4; 83, 58–150	77.4; 78, 42–113	79.9; 80, 42–150	0.002
Body mass index: Mean; median, range	31.0; 29.7, 21.0–49.0	32.8; 32.8, 19.0–45.0	32.1; 32.0, 19.0–49.0	0.013
Antecedents of nephropathy	2 (3.0%)	2 (1.7%)	4 (2.2%)	0.616
Antecedents of hepatopathy	0 (0%)	3 (2.5%)	3 (1.6%	0.553

Intergroup comparison values correspond to *p*-values using Mann–Whitney U for age, weight, and BMI and Fisher’s test for antecedents of nephropathy and hepatopathy.

**Table 2:** Acetaminophen pharmacokinetics in the whole study group.

Parameter	Mean; SD, Min–Max	Fold Range
C0 (μg/mL)	10.53; 5.23, 2.39–32.43	13.57
K10 (1/h)	0.48; 0.18, 0.19–1.44	7.58
t_1/2_ (h)	1.64; 0.54, 0.48–3.61	7.52
V (mg/(ug/mL))	118.61; 60.38, 30.83–339.11	11.00
CL (mg/(ug/mL)/h)	56.19; 35.31, 14.21–236.88	16.67
AUC 0–t (ug/mL*h)	20.58; 10.59, 4.12–57.66	14.00
AUC 0–inf (ug/mL*h)	24.18; 13.14, 4.22–70.35	16.67
AUMC (ug/mL*h^2^)	61.29; 45.4, 4.49–229.51	51.12
MRT (h)	2.36; 0.77, 0.70–5.20	7.43
Vss ((mg/(ug/mL))	118.61; 60.38, 30.83–339.11	11.00

Seven participants with antecedents of nephropathy or hepatopathy were removed from the study, leaving 186 valid participants. Abbreviations: SD, standard deviation; V, volume of distribution; CL, clearance; AUC, area under the plasma drug concentration-time curve; AUMC, area under the first moment curve; MRT, mean residence time; Vss, steady-state volume of distribution.

**Table 3:** Effect of sex in acetaminophen pharmacokinetics.

Parameter	Men (*n*= 66) (Mean; SD, Min–Max)	Women (*n* = 120) (Mean; SD, Min–Max)	Comparison (*p*-Value)
C0 (μg/mL)	8.29; 4.44, 2.39–26.70	11.77; 5.24, 3.31–32.43	<0.001
K10 (1/h)	0.45; 0.16, 0.19–0.93	0.49; 0.19, 0.20–1.44	0.207
t_1/2_ (h)	1.72; 0.58, 0.75–3.61	1.59; 0.51, 0.48–3.51	0.108
V (mg/(ug/mL))	146.33; 67.28, 37.45–339.11	103.83; 50.76, 30.83–302.39	<0.001
CL (mg/(ug/mL)/h)	65.88; 36.81, 22.17–205.77	50.87; 33.43, 14.21–236.88	<0.001
AUC 0–t (ug/mL*h)	16.18; 7.19, 4.74–32.13	23.00; 11.38, 4.12–57.66	<0.001
AUC 0–inf (ug/mL*h)	19.17; 8.59, 4.86–45.10	26.94; 14.38, 4.22–70.35	<0.001
AUMC (ug/mL*h^2^)	49.58; 31.44, 5.86–165.98	67.63; 50.46, 4.49–229.51	0.058
MRT (h)	2.49; 0.84, 1.08–5.20	2.30; 0.73, 0.70–5.07	0.108
Vss ((mg/(ug/mL))	146.33; 67.28, 37.45–339.11	103.83; 50.76, 30.83–302.39	<0.001

Abbreviations: SD, standard deviation; V, volume of distribution; CL, clearance; AUC, area under the plasma drug concentration-time curve; AUMC, area under the first moment curve; MRT, mean residence time; Vss, steady-state volume of distribution.

**Table 4:** Acetaminophen pharmacokinetics according to drug therapy received before the study initiation.

Parameter	No Substrates or Inhibitors (*n* = 83)	Treated with Substrates (*n* = 102)	Treated with Inhibitors (*n* = 5)
	Mean; SD, Range (*p*-Values: Crude; Adjusted by Sex)	Mean; SD, Range (*p*-Values: Crude; Adjusted by Sex)	Mean; SD, Range (*p*-Values: Crude; Adjusted by Sex)
C0 (μg/mL)	10.49; 4.90, 2.42–30.1 (reference)	10.63; 5.51, 2.39–32.43 (0.857; 0.998)	9.46; 3.67, 5.33–13.72 (0.644; 0.924)
K10 (1/h)	0.46; 0.17, 0.23–1.19 (reference)	0.49; 0.19, 0.19–1.44 (0.189; 0.207)	0.54; 0.21, 0.27–0.85 (0.318; 0.247)
t_1/2_ (h)	1.69; 0.52, 0.58–2.95 (reference)	1.59; 0.55, 0.48–3.61 (0.204; 0.227)	1.48; 0.64, 0.82–2.53 (0.374; 0.292)
V (mg/(ug/mL))	117.07; 59.46, 33.22–328.58 (reference)	119.18; 61.30, 30.83–339.11 (0.815; 0.660)	121.69; 52.18, 72.90–187.60 (0.865; 0.858)
CL (mg/(ug/mL)/h)	52.98; 30.78, 16.56–178.70 (reference)	58.54; 38.64, 14.21–236.88 (0.287; 0.230)	62.42; 27.07, 26.12–93.22 (0.503; 0.617)
AUC 0–t (ug/mL*h)	20.91; 9.84, 5.45–49.89 (reference)	20.41; 11.21, 4.12–57.66 (0.749; 0.606)	17.00; 7.58, 10.06–28.55 (0.384; 0.565)
AUC 0–inf (ug/mL*h)	24.87; 12.50, 5.60–60.39 (reference)	23.74; 13.69, 4.22–70.35 (0.562; 0.443)	19.64; 11.17, 10.73–38.29 (0.362; 0.522)
AUMC (ug/mL*h^2^)	64.48; 43.19, 6.46–178.09 (reference)	59.03, 47.28, 4.49–229.51 (0.417, 0.351)	48.53; 51.62, 19.21–139.91 (0.427; 0.538)
MRT (h)	2.44; 0.75, 0.84–4.26 (reference)	2.30; 0.79, 0.70–5.20 (0.204; 0.227)	2.13; 0.93, 1.18–3.65 (0.374; 0.292)
Vss ((mg/(ug/mL))	117.07; 59.46, 33.22–328.58 (reference)	119.18; 61.30, 30.83–339.11 (0.815; 0.660)	121.69; 52.18, 72.90–187.60 (0.865; 0.858)

The total number of participants is 186. Four participants received both substrates and inhibitors. Abbreviations: SD, standard deviation; V, volume of distribution; CL, clearance; AUC, area under the plasma drug concentration-time curve; AUMC, area under the first moment curve; MRT, mean residence time; Vss, steady-state volume of distribution.

**Table 5:** Acetaminophen pharmacokinetics according to drug therapy concomitant to acetaminophen.

Parameter	No Substrates or Inhibitors (*n* = 40)	Treated with Substrates (*n* = 146)	Other (Inhibitors + Inducers) (*n* = 1)
	Mean; SD, Range (*p*-Values: Crude; Adjusted by Sex)	Mean; SD, Range (*p*-Values: Crude; Adjusted by Sex)	
C0 (μg/mL)	12.10; 5.58, 3.04–30.10 (reference)	10.11; 5.07, 2.39–32.43 (0.032; 0.035)	3.34; -
K10 (1/h)	0.51; 0.25, 0.20–1.44 (reference)	0.47; 03.16, 0.19–0.96 (0.164; 0.177)	0.45; -
t_1/2_ (h)	1.60; 0.61, 0.48–3.51 (reference)	1.65; 0.52, 0.72–3.61 (0.657; 0.694)	1.54; -
V (mg/(ug/mL))	102.40; 55.64, 33.22–328.58 (reference)	123.11; 31.05, 30.83–339.11 (0.053; 0.054)	299.16; -
CL (mg/(ug/mL)/h)	51.48; 36.44, 14.21–178.70 (reference)	57.49; 35.01, 15.11–236.88 (0.340; 0.379)	134.34; -
AUC 0–t (ug/mL*h)	23.31; 11.35, 5.45–57.04 (reference)	19.83; 10.29, 4.2–57.66 (0.064; 0.072)	6.66; -
AUC 0–inf (ug/mL*h)	27.32; 14.45, 5.60–70.35 (reference)	23.32; 12.68, 4.22–66.19 (0.087; 0.099)	7.44; -
AUMC (ug/mL*h^2^)	70.42; 55.67, 4.94–224.94 (reference)	58.79; 42.03, 4.49–229.51 (0.150; 0.169)	16.58; -
MRT (h)	2.31; 0.88, 0.70–5.07 (reference)	2.38; 0.74, 1.04–5.20 (0.657; 0.694)	2.23; -
Vss ((mg/(ug/mL))	102.40; 55.64, 33.22–328.58 (reference)	123.11; 61.05, 30.83–339.11 (0.053; 0.054)	299.16; -

The total number of participants is 186. One participant received substrates, inhibitors, and inducers. Abbreviations: SD, standard deviation; V, volume of distribution; CL, clearance; AUC, area under the plasma drug concentration-time curve; AUMC, area under the first moment curve; MRT, mean residence time; Vss, steady-state volume of distribution.

**Table 6:** Acetaminophen pharmacokinetics according to *UGT1A6* genotypes.

Parameter	UGT1A6*1/*1 (*n* = 61)	UGT1A6*1/*Mutated (*n* = 79)	UGT1A6*Mutated/*Mutated (*n* = 35)
	Mean; SD, Range (*p*-Values: Crude; Adjusted by Sex)	Mean; SD, Range (*p*-Values: Crude; Adjusted by Sex)	Mean; SD, Range (*p*-Values: Crude; Adjusted by Sex)
C0 (μg/mL)	10.47; 4.98, 4.11–30.10 (reference)	10.06; 5.20, 2.39–26.70 (0.549; 0.710)	10.94; 5.09, 2.42–21.67 (0.669; 0.893)
K10 (1/h)	0.47; 0.17, 0.29–0.88 (reference)	0.47; 0.17, 0.23–0.96 (0.570; 0.466)	0.40; 0.13, 0.19–0.68 (0.019; 0.005)
t_1/2_ (h)	1.55; 0.45, 0.79–2.36 (reference)	1.64; 0.51, 0.72–2.95 (0.570; 0.339)	1.90; 0.64, 1.02–3.61 (0.019; 0.001)
V (mg/(ug/mL))	117.41; 52.96, 33.22–243.08 (reference)	127.26; 69.60, 37.45–339.11 (0.636; 0.400)	109.03; 54.73, 46.14–302.39 (0.400; 0.676)
CL (mg/(ug/mL)/h)	56.43; 29.86, 18.67–178.70 (reference)	59.42; 39.74, 14.21–236.88 (0.910; 0.687)	45.48; 26.39, 16.56–126.61 (0.081; 0.102)
AUC 0–t (ug/mL*h)	19.88; 9.58, 5.45–43.47 (reference)	19.56; 10.47, 4.12–57.04 (0.870; 0.942)	23.41; 10.95, 6.19–49.89 (0.113; 0.152)
AUC 0–inf (ug/mL*h)	22.99; 11.99, 5.60–53.57 (reference)	22.84; 12.75, 4.22–70.35 (0.910; 0.968)	28.96; 14.16, 7.90–60.39 (0.044; 0.045)
AUMC (ug/mL*h^2^)	55.53; 39.99, 7.61–160.47 (reference)	56.90; 42.67, 4.49–229.51 (0.658; 0.760)	83.77; 54.66, 17.14–224.94 (0.010; 0.006)
MRT (h)	2.24; 0.65, 1.13–3.41 (reference)	2.36; 0.73, 1.04–4.26 (0.570; 0.339)	2.75; 0.93, 1.48–5.20 (0.019; 0.001)
Vss ((mg/(ug/mL))	117.41; 52.96, 33.22–243.08 (reference)	127.26; 69.60, 37.45–339.11 (0.636; 0.400)	109.03, 54.73, 46.14–302.39 (0.400; 0.676)

The number of participants was 175 because 11 participants carrying UGT1A9 variant alleles were excluded to avoid confounders. Mutated genes include UGT1A6*2, *3, *4, and *9. No other variant alleles were observed in the study group. Abbreviations: SD, standard deviation; V, volume of distribution; CL, clearance; AUC, area under the plasma drug concentration-time curve; AUMC, area under the first moment curve; MRT, mean residence time; Vss, steady-state volume of distribution.

**Table 7:** Acetaminophen pharmacokinetics according to *UGT1A6* diplotypes.

Parameter	UGT1A6*1/*1 (*n* = 61)	UGT1A6*1/*2 (*n* = 59)	UGT1A6*1/*3 (*n* = 14)	UGT1A6*1/*other (*n* = 6)	UGT1A6*2/*2 (*n* = 15)	UGT1A6*2/*3 (*n* = 8)	UGT1A6*2/*4 (*n* = 8)	UGT1A6*3/*4 (*n* = 4)
	Mean; SD, Range (*p*-Values: Crude; Adjusted by Sex)	Mean; SD, Range (*p*-Values: Crude; Adjusted by Sex)	Mean; SD, Range (*p*-Values: Crude; Adjusted by Sex)	Mean; SD, Range (*p*-Values: Crude; Adjusted by Sex)	Mean; SD, Range (*p*-Values: Crude; Adjusted by Sex)	Mean; SD, Range (*p*-Values: Crude; Adjusted by Sex)	Mean; SD, Range (*p*-Values: Crude; Adjusted by Sex)	Mean; SD, Range (*p*-Values: Crude; Adjusted by Sex)
C0 (μg/mL)	10.47; 4.98, 4.11–30.10 (reference)	9.86; 5.54, 2.39–26.70 (0.364; 0.631)	11.25; 4.28, 5.97–22.00 (0.602; 0.456)	9.29; 3.58, 3.85–13.65 (0.574; 0.329)	10.39; 5.93, 2.42–21.67 (0.961; 0.749)	10.72; 4.05, 6.19–18.53 (0.895; 0.642)	11.62; 4.96, 6.23–21.12 (0.541; 0.716)	11.90; 5.78, 7.20–20.07 (0.583; 0.954)
K10 (1/h)	0.49; 0.17, 0.29–0.88 (reference)	0.47; 0.18, 0.23–0.96 (0.307; 0.454)	0.49; 0.12, 0.25–0.70 (0.511; 0.947)	0.47; 0.08, 0.31–0.52 (0.797; 0.692)	0.36; 0.11, 0.19–0.55 (0.004; 0.004)	00.46; 0.13, 0.27–0.68 (0.952; 0.642)	00.37; 0.11, 0.25–0.53 (0.038; 0.037)	0.51; 0.12, 0.39–0.65 (0.484; 0.849)
t_1/2_ (h)	1.55; 0.45, 0.79–2.36 (reference)	1.67; 0.53, 0.72–2.95 (0.307; 0.202)	1.52; 0.46, 0.98–2.77 (0.511; 0.804)	1.53; 0.33, 1.34–2.21 (0.797; 0.913)	2.14; 0.73, 1.25–3.61 (0.004; 0.004)	1.62; 0.49, 1.02–2.55 (0.952; 0.732)	2.03; 0.57, 1.30–2.75 (0.038; 0.006)	1.41; 0.32, 1.07–1.76 (0.484; 0.545)
V (mg/(ug/mL))	117.41; 52.96, 33.22–243.08 (reference)	133.38; 74.72, 37.45–339.11 (0.451; 0.204)	100.59; 36.77, 45.45–167.38 (0.483; 0.186)	128.95; 70.07, 73.26–259.56 (0.656; 0.419)	121.02; 75.23, 46.14–302.39 (0.815; 0.578)	105.14; 38.09, 53.98–161.56 (0.660; 0.348)	99.11; 37.67, 47.34–160.64 (0.402; 0.462)	97.69; 38.99, 49.83–138.90 (0.541; 0.850)
CL (mg/(ug/mL)/h)	56.43; 29.86, 18.67–178.70 (reference)	62.62; 44.53 14.21–236.88 (0.922; 0.441)	47.07; 16.32, 29.43–77.45 (0.280; 0.226)	56.29; 19.82, 37.28–81.59 (0.991; 0.838)	46.63; 31.87, 20.68–126.61 (0.129; 0.335)	50.30; 25.59, 21.61–88.59 (0.583; 0.468)	34.65; 11.09, 16.56–50.26 (0.031; 0.056)	53.45; 31.26; 19.62–90.03 (0.848; 0.859)
AUC 0–t (ug/mL*h)	19.88; 9.58, 5.45–43.47 (reference)	19.46; 11.58, 4.12–57.04 (0.667; 0.968)	20.96; 6.50, 12.34–32.13 (0.369; 0.549)	17.50; 6.01, 9.71–24.72 (0.640; 0.311)	22.48; 10.72, 6.19–40.83 (0.340; 0.476)	22.06; 11.00, 10.62–40.03 (0.590; 0.351)	26.51; 10.97, 17.70–49.89 (0.072; 0.096)	23.13; 14.92, 10.67–43.86 (0.771; 0.961)
AUC 0–inf (ug/mL*h)	22.99; 11.99, 5.60–53.57 (reference)	23.03; 14.21, 4.22–70.35 (0.922; 0.845)	23.56; 7.41, 12.91–33.98 (0.410; 0.720)	219.50; 6.00, 12.26–26.83 (0.761; 0.268)	29.35; 14.06, 7.90–48.35 (0.129; 0.108)	26.16; 14.86, 11.29–46.28 (0.576; 0.322)	32.55; 13.79, 19.90–60.39 (0.031; 0.050)	25.98; 17.97, 11.11–50.98 (0.907; 0.921)
AUMC (ug/mL*h^2^)	55.33; 39.99 7.61–160.47 (reference)	59.43; 47.47, 4.49–229.51 (0.748; 0.512)	52.94; 26.40, 20.68–121.42 (0.531; 0.941)	41.79; 10.02, 23.76–52.72 (0.981; 0.259)	91.81; 57.47, 25.76–224.94 (0.017; 0.007)	68.74; 56.02, 19.94–165.98 (0.646; 0.294)	97.35; 52.30, 43.67–178.09 (0.011; 0.010)	58.49; 51.00, 17.14–129.50 (0.930; 0.769)
MRT (h)	2.24; 0.65, 1.13–3.41 (reference)	2.42; 0.77, 1.04–4.26 (0.307; 0.202)	2.20; 0.66, 1.42–4.00 (0.511; 0.804)	2.21; 0.48, 1.93–3.18 (0.797; 0.913)	3.18; 1.05, 1.81–5.20 (0.004; <0.001)	2.33; 0.71, 1.48–3.68 (0.925; 0.732)	2.93; 0.82, 1.88–3.97 (0.038; 0.006)	2.03; 0.46, 1.54–2.54 (0.484; 0.545)
Vss ((mg/(ug/mL))	117.41; 52.96, 33.22–243.08 (reference)	133.38; 74.72, 37.45–339.11 (0.451; 0.204)	100.59; 36.77, 45.45–167.38 (0.483; 0.186)	128.95; 70.07, 73.26–259.56 (0.656; 0.419)	121.02; 75.23, 46.14–302.39 (0.815; 0.587)	105.14; 38.09, 53.98–161.56 (0.660; 0.348)	99.11; 37.67, 47.34–160.64 (0.402; 0.462)	97.69; 38.99, 49.83–138.90 (0.541; 0.850)

The number of participants was 175 because 11 participants carrying UGT1A9 variant alleles were excluded to avoid confounders. Mutated genes include UGT1A6*2, *3, *4, and *9. No other variant alleles were observed in the study group. Abbreviations: SD, standard deviation; V, volume of distribution; CL, clearance; AUC, area under the plasma drug concentration-time curve; AUMC, area under the first moment curve; MRT, mean residence time; Vss, steady-state volume of distribution.

## Data Availability

All data generated or analyzed during this study are included in this published article.

## Supplementary Materials

The following supporting information can be downloaded at: https://www.mdpi.com/article/10.3390/jpm12050720/s1, Table S1: Drug therapy received within two weeks before the study initiation; Table S2: Concomitant drug therapy received after the study initiation; Table S3: Details of the TaqMan Assays used in this study; Table S4: Influence of body mass index (BMI) in acetaminophen pharmacokinetics; Table S5: Acetaminophen pharmacokinetics according to UGT1A9 diplotypes; Table S6: Acetaminophen pharmacokinetics depending on the need for additional painkiller therapy; Table S7: UGT1A6 and UGT1A9 genotypes according to the need for additional painkiller therapy.
