# Supplementary material for: Common UGT1A6 Variant Alleles Determine Acetaminophen Pharmacokinetics in Man
Source: J Pers Med. 2022 Apr 29;12(5):720. doi: 10.3390/jpm12050720 (PMC9143054; doi:10.3390/jpm12050720)
Supplement: Supplementary file 1 [file jpm-12-00720-s001.zip › jpm-1669939-supplementary.pdf]

Journal of Personalized Medicine

MDPI

Supplementary Materials

Table S1. Drug therapy received within two weeks before the study initiation.

Drug	Median Dose (mg/24 h)	Administration Route	Total of Participants (*n*)	Men Who Received the Drug	Men Who Did Not Receive the Drug	Women Who Received the Drug	Women Who Did Not Receive the Drug	Intergroup Comparison Values: *p*-Value (Chi-Square)
Omeprazole	20	oral	40	11	55	29	91	0,234
ASA	100	oral	28	13	53	15	105	0,189
Hydrochlorothiazide	25	oral	24	7	59	17	103	0,489
Aceclofenac	100	oral	18	1	65	17	103	**0,005**
Lorazepam	1	oral	15	1	65	14	106	**0,015**
Acetaminophen	3000	oral	14	5	61	9	111	0,985
Simvastatin	80	oral	14	6	60	8	112	0,549
Ibuprofen	1200	oral	15	2	64	13	107	0,062
Atorvastatin	80	oral	12	5	61	7	113	0,643
Pantoprazole	40	oral	12	4	62	8	112	0,872
Tramadol	100	oral	12	3	63	9	111	0,433
Torasemide	20	oral	11	5	61	6	114	0,477
Amlodipine	10	oral	10	3	63	7	113	0,709
Enalapril	10	oral	10	3	63	7	113	0,709
Calcium	1000	oral	9	3	63	6	114	0,890
Diclofenac	100	oral	9	2	64	7	113	0,394
Doxazosin	4	oral	9	8	58	1	119	**0,001**
Heparin	40	subcutaneous	9	2	64	7	113	0,394
Furosemide	40	oral	9	2	64	7	113	0,394
Metamizole	1725	oral	9	3	63	6	114	0,890
Valsartan	80	oral	9	3	63	6	114	0,890
Alprazolam	1.5	oral	8	1	65	7	113	0,165
Irbesartan	150	oral	8	4	62	4	116	0,380
Metformin	1500	oral	8	3	63	5	115	0,903
Acenocoumarol	2	oral	7	5	61	2	118	**0,043**
Bisoprolol	5	oral	7	2	64	5	115	0,697
Candesartan	8	oral	7	2	64	5	115	0,697
Carvedilol	25	oral	7	2	64	5	115	0,697

Atenolol	100	oral	6	2	64	4	116	0,911
Chondroitin sulfate	800	oral	6	1	65	5	115	0,327
Gliclazide	30	oral	6	2	64	4	116	0,911
Glucosamine	1500	oral	6	0	66	6	114	0,065
Insulin	100U/ml	subcutaneous	7	2	64	5	115	0,698
Losartan	50	oral	6	1	65	5	115	0,327
Paroxetine	20	oral	6	0	66	6	114	0,065
Pentoxifylline	800	oral	6	4	62	2	118	0,105
Sertraline	50	oral	6	1	65	5	115	0,327
Verapamil	360	oral	6	3	63	3	117	0,450
Zolpidem	10	oral	6	1	65	5	115	0,327
Alendronic acid	10	oral	5	2	64	3	117	0,831
Amiloride	5	oral	5	1	65	4	116	0,463
Fluoxetine	20	oral	5	0	66	5	115	0,093
Levothyroxine	0.025	oral	5	1	65	4	116	0,463
Trazodone	150	oral	5	2	64	3	117	0,831
Clorazepic acid	5	oral	4	1	65	3	117	0,658
Eprosartan	600	oral	4	2	64	2	118	0,540
Fluticasone	0.500	inhalation	4	0	66	4	116	0,134
Iron	80	oral	4	1	65	4	116	0,464
Nifedipine	40	oral	4	1	65	3	117	0,658
Pravastatin	40	oral	4	0	66	4	116	0,134
Ramipril	2.5	oral	4	2	64	2	118	0,540
Risedronic acid	5	oral	4	0	66	4	116	0,134
Tamsulosin	0.4	oral	6	6	60	0	120	**0,001**
Trimetazidine	60	oral	4	0	66	4	116	0,134
Allopurinol	100	oral	3	2	64	1	119	0,255
Bromazepam	9	oral	3	1	65	2	118	0,937
Captopril	50	oral	3	2	64	1	119	0,255
Diazepam	10	oral	3	1	65	2	118	0,937
Escin	100	oral	3	0	66	3	117	0,195
Dutasteride	0.500	oral	3	3	63	0	120	**0,019**
Fentanyl	8.3	transdermal	3	0	66	3	117	0,195
Gabapentin	900	oral	3	0	66	3	117	0,195
Lansoprazole	15	oral	3	1	65	2	118	0,937
Salmeterol	0.100	inhalation	3	0	66	3	117	0,195
Telmisartan	40	oral	3	0	66	3	117	0,195
Tiotropium	0.005	inhalation	3	3	63	0	120	**0,019**

Trifusal	600	oral	3	0	66	3	117	0,195
Troxerutin	1000	oral	3	0	66	3	117	0,195
Venlafaxine	75	oral	3	0	66	3	117	0,195
Amitriptyline	50	oral	2	1	65	1	119	0,666
Cefditoren	400	oral	2	1	65	1	119	0,666
Cyclobenzaprine	30	oral	2	0	66	2	118	0,292
Citalopram	20	oral	2	1	65	1	119	0,666
Clonazepam	3	oral	2	0	66	2	118	0,292
Deflazacort	30	oral	2	1	65	1	119	0,666
Dexamethasone	30	oral	2	0	66	2	118	0,292
Dexketoprofen	75	oral	2	2	64	0	120	0,055
Diacerein	100	oral	2	1	65	1	119	0,666
Diltiazem	180	oral	2	1	65	1	119	0,666
Dobesilic acid	1500	oral	2	0	66	2	118	0,292
Donepezil	10	oral	2	1	65	1	119	0,666
Hidrosmin	600	oral	2	0	66	2	118	0,292
Hydroxyzine	75	oral	2	0	66	2	118	0,292
Indapamide	1.5	oral	2	0	66	2	118	0,292
Lisinopril	20	oral	2	0	66	2	118	0,292
Lovastatin	20	oral	2	0	66	2	118	0,292
Plantago seed	11000	oral	2	0	66	2	118	0,292
Raloxifene	60	oral	2	0	66	2	118	0,292
Salbutamol	0.200	inhalation	2	2	64	0	120	0,055
Sulpiride	150	oral	2	0	66	2	118	0,292
Tetrazepam	100	oral	2	0	66	2	118	0,292
Troxerutin	1000	oral	2	0	66	2	118	0,292
Almagate	3000	oral	1	0	66	1	119	0,457
Altizide	30	oral	1	0	66	1	119	0,457
Amiodarone	600	oral	1	0	66	1	119	0,457
Azathioprine	140	oral	1	1	65	0	120	0,176
Betahistine	24	oral	1	0	66	1	119	0,457
Biperiden	6	oral	1	1	65	0	120	0,176
Bosentan	250	oral	1	0	66	1	119	0,457
Budesonide	0.400	inhalation	1	1	65	0	120	0,176
Buprenorphine	0.875	transdermal	1	0	66	1	119	0,457
Carbidopa	75	oral	1	1	65	0	120	0,176
Cetirizine	10	oral	1	0	66	1	119	0,457
Diflunisal	1000	oral	1	1	65	0	120	0,176

Domperidone	30	oral	1	0	66	1	119	0,457
Enoxaparin	40	subcutaneous	1	1	65	0	120	0,176
Escitalopram	10	oral	1	1	65	0	120	0,176
Esomeprazole	40	oral	1	0	66	1	119	0,457
Estradiol	0.017	transdermal	1	0	66	1	119	0,457
Etidronic acid	800	oral	1	0	66	1	119	0,457
Etoricoxib	30	oral	1	1	65	0	120	0,176
Felodipine	5	oral	1	1	65	0	120	0,176
Fexofenadine	180	oral	1	0	66	1	119	0,457
Fluvoxamine	50	oral	1	0	66	1	119	0,457
Fosinopril	10	oral	1	0	66	1	119	0,457
Gemfibrozil	900	oral	1	0	66	1	119	0,457
Glimepiride	1	oral	1	1	65	0	120	0,176
Glutamic acid	5000	oral	1	1	65	0	120	0,176
Halazepam	60	oral	1	0	66	1	119	0,457
Indomethacin	50	oral	1	1	65	0	120	0,176
Ipratropium	0.750	inhalation	1	1	65	0	120	0,176
Ketorolac	40	oral	1	1	65	0	120	0,176
Lactulose	20	oral	1	1	65	0	120	0,176
Lercanidipine	10	oral	1	0	66	1	119	0,457
Levodopa	300	oral	1	1	65	0	120	0,176
Loprazolam	1	oral	1	0	66	1	119	0,457
Lormetazepam	1	oral	1	0	66	1	119	0,457
Maprotiline	75	oral	1	0	66	1	119	0,457
Meloxicam	7.5	oral	1	0	66	1	119	0,457
Mesalazine	2000	oral	1	0	66	1	119	0,457
Metimazole	30	oral	1	0	66	1	119	0,457
Metoprolol	100	oral	1	1	65	0	120	0,176
Migitol	150	oral	1	1	65	0	120	0,176
Mirtazapine	30	oral	1	0	66	1	119	0,457
Misoprostol	0.400	oral	1	0	66	1	119	0,457
Naproxen	550	oral	1	0	66	1	119	0,457
Nevibolol	5	oral	1	0	66	1	119	0,457
Nimodipine	360	oral	1	0	66	1	119	0,457
Nitroglycerin	4.8	sublingual	1	1	65	0	120	0,176
Nitroglycerin	5	transdermal	1	0	66	1	119	0,457
Olmesartan	10	oral	1	1	65	0	120	0,176
Orlistat	160	oral	1	1	65	0	120	0,176

Piroxicam	20	oral	1	0	66	1	119	0,457
Prednisone	5	oral	1	1	65	0	120	0,176
Pregabalin	150	oral	1	0	66	1	119	0,457
Propafenone	450	oral	1	1	65	0	120	0,176
Propranolol	80	oral	1	0	66	1	119	0,457
Quinapril	20	oral	1	1	65	0	120	0,176
Rabeprazole	10	oral	1	1	65	0	120	0,176
Repaglinide	0.500	oral	1	1	65	0	120	0,176
Rupatadine	10	oral	1	0	66	1	119	0,457
Sotalol	80	oral	1	0	66	1	119	0,457
Spironolactone	50	oral	1	0	66	1	119	0,457
Tenoxicam	20	oral	1	0	66	1	119	0,457
Temazepam	50	oral	1	0	66	1	119	0,457
Ticlopidine	500	oral	1	1	65	0	120	0,176
Thiethylperazine	19.5	oral	1	0	66	1	119	0,457
Tolterodine	4	oral	1	0	66	1	119	0,457
Tranexamic acid	1000	oral	1	1	65	0	120	0,176
Triazolam	0.250	oral	1	0	66	1	119	0,457
Triamterene	25	oral	1	0	66	1	119	0,457
Ursodeoxycholic acid	500	oral	1	1	65	0	120	0,176
Valproic acid	750	oral	1	0	66	1	119	0,457

Statistically significant values are marked with bold. Abbreviations: ASA, acetylsalicylic acid.

Table S2. Concomitant drug therapy received after the study initiation.

Drug	Median Dose (mg/24 h)	Administration Route	Total of Participants (*n*)	Men Who Received the Drug	Men Who Did Not Receive the Drug	Women Who Received the Drug	Women Who Did Not Receive the Drug	Intergroup Comparison Values: *p*-Value (Chi-Square)
Pantoprazole	40	intravenous	186	66	0	120	0	1.000
Ondansetron	12	intravenous	185	64	2	120	0	0.055
Metamizole	6000	intravenous	176	61	5	115	5	0.324
Gentamicin	350	intravenous	173	60	6	113	7	0.404
Cefazolin	3000	intravenous	164	57	9	107	13	0.571
Morphine Chloride	10	subcutaneous	128	47	19	81	39	0.601
Dalteparin	2500 UI	subcutaneous	121	38	28	83	37	0.113
Ketorolac	90	intravenous	79	28	38	51	69	0.992
Tramadol	300	intravenous	54	20	46	34	86	0.777
Insulin	variable	subcutaneous	51	19	47	32	88	0.756
Dexamethasone	4	intravenous	31	10	56	21	99	0.158
Vancomycin	2000	intravenous	18	6	60	12	108	0.841
Ropivacaine	150	epidural	7	3	63	4	116	0.678
Enoxaparin	40	subcutaneous	6	4	62	2	118	0.106
Iron	80	oral	6	0	66	6	114	0.065
Fentanyl	2.8	intravenous	4	1	65	3	117	0.658
Furosemide	40	intravenous	4	1	65	3	117	0.658
Atropine	0.45	intravenous	2	2	64	0	120	0.055
Metoclopramide	30	intravenous	2	1	65	1	119	0.666
ASA	100	oral	1	1	65	0	120	0.176
Acyclovir	1050	intravenous	1	1	65	0	120	0.176
Alprazolam	0.500	oral	1	0	66	1	119	0.457
Amiodarone	600	intravenous	1	1	65	0	120	0.176
Amoxicillin / Clavulanic acid	3000 / 600	intravenous	1	0	66	1	119	0.457
Atorvastatin	80	oral	1	1	65	0	120	0.176
Bromazepam	4.5	oral	1	1	65	0	120	0.176
Carbamazepine	800	oral	1	1	65	0	120	0.176
Carvedilol	25	oral	1	1	65	0	120	0.176
Ceftriaxone	4000	intravenous	1	1	65	0	120	0.176
Dopamine	345	intravenous	1	1	65	0	120	0.176
Haloperidol	20	intravenous	1	1	65	0	120	0.176
Isoniazid	300	intravenous	1	1	65	0	120	0.176

Lactulose	30000	oral	1	1	65	0	120	0.176
Midazolam	190	intravenous	1	1	65	0	120	0.176
Nitroglycerin	28	intravenous	1	1	65	0	120	0.176
Phenytoin	300	intravenous	1	1	65	0	120	0.176
Pyrazinamide	1800	oral	1	1	65	0	120	0.176
Pyridoxine	300	intravenous	1	1	65	0	120	0.176
Propofol 2%	5760	intravenous	1	1	65	0	120	0.176
Rifampicin	600	oral	1	1	65	0	120	0.176
Thiamine	100	intravenous	1	1	65	0	120	0.176
Trazodone	100	oral	1	1	65	0	120	0.176
Valproic acid	800	intravenous	1	1	65	0	120	0.176

Statistically significant values are marked with bold. Abbreviations: ASA, acetylsalicylic acid.

Table S3. Details of the Taqman Assays used in this study.

Allele	Random SNV ID	Assay Identification (Thermo Scientific, Alcobendas, Spain)	Genotyping Results (Non-Mutated, Heterozygous, Homozygous)	Minor Allele Frequency in This Study	Hardy Weinberg Equilibrium (Pearson)	Minor Allele Frequency in GnomAD for Southern Europeans
*UGT1A1*6*	rs4148323 G>A	C____559715_20	186, 0, 0	0.000	-	0.001
*UGT1A1*27*	rs35350960 C>A	C___2307598_20	186, 0, 0	0.000	-	0.000
*UGT1A6*2, *3, and *4*	rs6759892 T>G	C___1432204_30	66, 85, 35	0.4167	0.414	0.4175
*UGT1A6*2, *5, and *8*	rs2070959 A>G	C__15868110_30	87, 84, 15	0.3064	0.395	0.3478
*UGT1A6*2, *4, *8, and *9*	rs1105879 A>C	C___1173642_30	81, 82, 23	0.3441	0.751	0.3737
*UGT1A6*2, *3b, and *4*	rs1105880 A>G	C___1173641_10	66, 85, 35	0.4167	0.414	0.3747
*UGT1A9*3*	rs72551330 T>C	C__64627083_10	175, 10, 1	0.0323	0.059	0.0139

Table S4. Influence of body mass index (BMI) in acetaminophen pharmacokinetics.

Parameter	Low/Normal Weight, BMI < 25.0, (*n* = 16) (Mean; SD, Min–Max)	Overweight, BMI 25.0–29.9 (*n* = 59) (Mean; SD, Min–Max)	Obesity, BMI ≥ 30.0 (*n* = 111) (Mean; SD, Min–Max)	Comparison Low/Normal vs. Overweight	Comparison Low/Normal vs. Obesity	Comparison Overweight vs. Obesity
C0 (μg/ml)	11.25; 7.13, 2.42-30.10	10.71; 5.95, 2.39-32.43	10.34; 4.51, 2.95-23.43	0.979	0.626	0.835
K10 (1/h)	0.42; 0.15, 0.25-0.84	0.50; 0.18, 0.20-0.96	0.47; 0.19, 0.19-1.44	0.100	0.251	0.378
$t_{1/2}$ (h)	1.80; 0.51, 0.83-2.77	1.58; 0.56, 0.72-3.51	1.64; 0.52, 0.48-3.61	0.165	0.258	0.498
V (mg/(ug/ml))	111.31; 56.87, 33.22-214.04	118.75; 60.95, 30.83-328.58	119.53; 61.00, 42.68-339.11	0.754	0.638	0.979
CL (mg/(ug/ml)/h)	49.85; 31.05, 22.49-126.61	59.08; 40.69, 16.06-236.88	55.57; 32.89, 14.21-178.70	0.345	0.387	0.688
AUC 0–t (ug/ml*h)	22.39; 10.08, 6.19-35.33	19.96; 10.47, 4.12-57.66	20.65; 10.78, 5.38-57.04	0.345	0.391	0.787
AUC 0–inf (ug/ml*h)	26.37; 11.81, 7.90-44.47	23.18; 12.60, 4.22-62.26	24.20; 13.66, 5.60-70.35	0.345	0.387	0.688
AUMC (ug/ml*h^2)	68.80; 39.53, 23.03-148.88	56.60; 44.42, 4.49-224.94	62.70; 46.81, 4.94-229.51	0.170	0.302	0.431
MRT (h)	2.60; 0.74, 1.19-4.00	2.29; 0.81, 1.04-5.07	2.37; 0.76, 0.70-5.20	0.165	0.258	0.498
Vss ((mg/(ug/ml))	111.31; 56.87, 33.22-214.04	118.75; 60.95, 30.83-328.58	119.53; 61.00, 42.68-339.11	0.754	0.638	0.979

Abbreviations: SD, standard deviation; V, volume of distribution; CL, clearance; AUC, area under the plasma drug concentration-time curve; AUMC, area under the first moment curve; MRT, mean residence time; Vss, Steady-state volume of distribution.

Table S5. Acetaminophen pharmacokinetics according to *UGT1A9* diplotypes.

Parameter	***UGT1A9*1/*1*** (*n* = 60) Mean; SD, Range (*p*-Values: Crude; Adjusted by Sex)	***UGT1A9*1/*3*** (*n* = 4) Mean; SD, Range (*p*-Values: Crude; Adjusted by Sex)	***UGT1A9*3/*3*** (*n* = 1)
C0 (μg/ml)	10.47; 4.98, 4.11-30.10 (reference)	8.46; 4.05, 4.58-12.07 (0.433; 0.537)	6.67
K10 (1/h)	0.49; 0.17, 0.29-0.88 (reference)	0.55; 0.13, 0.45-0.71 (0.221; 0.493)	0.39
$t_{1/2}$ (h)	1.55; 0.45, 0.79-2.36 (reference)	1.31; 0.29, 0.98-1.56 (0.221; 0.294)	1.78
V (mg/(ug/ml))	117.41; 52.96, 33.22-243.08 (reference)	143.29; 69.99, 82.82-218.25 (0.351; 0.439)	149.83
CL (mg/(ug/ml)/h)	56.43; 29.86, 18.67-178.70 (reference)	81.33; 52.14, 37.19-153.89 (0.130; 0.153)	58.35
AUC 0–t (ug/ml*h)	19.88; 9.58, 5.45-43.47 (reference)	14.85; 8.09, 5.95-24.04 (0.281; 0.381)	14.69
AUC 0–inf (ug/ml*h)	22.99; 11.99, 5.60-53.57 (reference)	16.27; 8.92, 6.50-26.89 (0.294; 0.338)	17.14
AUMC (ug/ml*h^2)	55.33; 39.99, 7.61-160.47 (reference)	32.21; 21.00, 9.22-59.88 (0.210; 0.309)	44.00
MRT (h)	2.24; 0.65, 1.13-3.41 (reference)	1.89; 0.41, 1.42-2.25 (0.221; 0.294)	2.57
Vss ((mg/(ug/ml))	117.41; 52.96, 33.22-243.08 (reference)	143.29; 69.99, 80.82-218.25 (0.351; 0.439)	149.83

Abbreviations: SD, standard deviation; V, volume of distribution; CL, clearance; AUC, area under the plasma drug concentration-time curve; AUMC, area under the first moment curve; MRT, mean residence time; Vss, Steady-state volume of distribution.

Table S6. Acetaminophen pharmacokinetics depending on the need for additional painkiller therapy.

Parameter	No Painkillers Besides Acetaminophen (*n* = 1)	Additional NSAID Therapy (*n* = 38) Mean; SD, Range (*p*-Values: Crude; Adjusted by Sex)	Opiate Therapy (*n* = 147) Mean; SD, Range (*p*-Values: Crude; Adjusted by Sex)
C0 (μg/ml)	3.34	11.87; 5.70, 3.04-30.10 (reference)	10.24; 5.05, 2.39-32.43 (0.085; 0.058)
K10 (1/h)	0.45	0.52; 0.25, 0.26-1.44 (reference)	0.46; 0.16, 0.19-0.96 (0.066-0.062)
$t_{1/2}$ (h)	1.54	1.54; 0.53, 0.48-2.66 (reference)	1.66; 0.54, 0.72-3.61 (0.212; 0.199)
V (mg/(ug/ml))	299.16	105.18; 56.74, 33.22-328.58 (reference)	120.88; 59.39, 30.83-339.11 (0.143; 0.096)
CL (mg/(ug/ml)/h)	134.34	53.80; 36.42, 14.21-178.70 (reference)	56.28; 34.65, 15.11-236.88 (0.697; 0.657)
AUC 0–t (ug/ml*h)	6.66	22.30; 11.22, 5.45-57.04 (reference)	20.23; 10.39, 4.12-57.66 (0.282; 0.229)
AUC 0–inf (ug/ml*h)	7.44	25.74; 13.97, 5.60-70.35 (reference)	23.89; 12.91, 4.22-66.19 (0.439; 0.382)
AUMC (ug/ml*h^2)	16.58	62.83; 48.77, 4.94-211.24 (reference)	61.19; 44.66, 4.49-229.51 (0.843; 0.805)
MRT (h)	2.23	2.22; 0.76, 0.70-3.84 (reference)	2.40; 0.78, 1.04-5.20 (0.212; 0.199)
Vss ((mg/(ug/ml))	299.16	105.18; 56.74, 33.22-328.58 (reference)	120.88; 59.39, 30.83-339.11 (0.143; 0.096)

Abbreviations: SD, standard deviation; V, volume of distribution; CL, clearance; AUC, area under the plasma drug concentration-time curve; AUMC, area under the first moment curve; MRT, mean residence time; Vss, Steady-state volume of distribution.

Table S7. *UGT1A6* and *UGT1A9* genotypes according to the need for additional painkiller therapy.

**Genotypes/diplotypes**	**Additional NSAID Therapy** ***n*, %** **(*p*-Values: Crude; Adjusted by Sex)**	**Opiate Therapy** ***n*, %** **(*p*-Values: Crude; Adjusted by Sex)**
***UGT1A6* diplotypes**	***n* =34**	***n* = 140**
*UGT1A6*1/*1*	12, 35.3% (reference)	49, 35.0% (0.765, 0.764)
*UGT1A6*1/*2*	10, 29.4% (reference)	49, 35.0% (0.569, 0.558)
*UGT1A6*1/*3*	5, 14.7% (reference)	8, 5.7% (0.083, 0.085)
*UGT1A6*1/*other*	1, 2.9% (reference)	5, 3.6% (0.891, 0.909)
*UGT1A6*2/*2*	1, 2.9% (reference)	14, 10.0% (0.197, 0.201)
*UGT1A6*2/*3*	1, 2.9% (reference)	7, 5.0% (0.625, 0.611)
*UGT1A6*2/*4*	3, 8.8% (reference)	5, 3.6% (0.891, 0.909)
*UGT1A6*3/*4*	1, 2.9% (reference)	3, 2.1% (0.756, 0.726)
***UGT1A6* genotypes**	***n* = 34**	***n* = 140**
*UGT1A6*1/*1*	12, 35.3% (reference)	49, 35.0% (0.765, 0.764)
*UGT1A6*1/mut*	16, 47.0 (reference)	62, 44.3% (0.661, 0.672)
*UGT1A6*mut/*mut*	6, 19.4% (reference)	29, 20.7% (0.848, 0.864)
***UGT1A9* diplotypes**	***n* = 12**	***n* = 53**
*UGT1A9*1/*1*	10, 83.3% (reference)	48, 90.6% (0.892, 0.891)
*UGT1A9*1/*3*	1, 8.3% (reference)	5, 9.4% (0.203, 0.203)
*UGT1A9*3/*3*	1, 8.3% (reference)	0, 0.0% (0.060, 0.059)

The genotypes/diplotypes categories are marked in bold. One participant out of the 186 was not included because did not receive any painkiller therapy besides acetaminophen. *UGT1A6* diplotypes and genotypes corresponded only to individuals without *UGT1A9* gene variants and vice-versa. NSAID, Non-steroidal anti-inflammatory drugs.
